# Deconstructing the Cognitive Estimation Task: A Developmental Examination and Intra-Task Contrast

**DOI:** 10.1038/srep39316

**Published:** 2016-12-19

**Authors:** Sarit Silverman, Sarit Ashkenazi

**Affiliations:** 1 Learning Disabilities Program, The Seymour Fox School of Education, The Hebrew University of Jerusalem, Mount Scopus Jerusalem 91905, Israel.

## Abstract

The cognitive estimation task (CET) requires participants to answer estimation questions that lack definitive answers. Few studies examine CET performance in healthy populations, and even fewer in children. Previous research has not considered differences between categories within the CET. The categories differ in their reliance on units of measurement, which is significant when examining CET performance in children due to educational factors. The goal of the study was to examine CET performance in 10 and 12 year-old children and contrast the CET categories. We found a developmental effect in overall CET performance: children’s performance was more extreme than adults but no differences were found between the groups of children. Examination of the CET categories revealed differential developmental trajectories: the children’s scores were more extreme in weight and time, while comparable to adults in quantity. We conclude that CET questions that require application of units of measurement are more difficult for childern due to higher involvement of executive functions, and children have less experience applying them in daily life. The CET is not a unified construct and has the potential to shed light on how children acquire an understanding of magnitudes and units of measurement.

Cognitive estimation questions require participants to provide a reasonable response when a specific answer is not available^1^. This contrasts with general knowledge questions, which have one exact answer that is readily available and easily retrievable. For example, the cognitive estimation question, “how many seeds are there in a watermelon”^2^? the solution is drawn indirectly from personal experience; most participants have eaten watermelon but have never counted the number of seeds. Beyond accessing the relevant semantic framework, the solution of cognitive estimation questions involves several components of executive functions (EF) including: planning, problem solving, cognitive flexibility, working memory, mental control, inhibition, self-monitoring and self-correction^2^,^3^.

EF is a set of high-order cognitive processes that play a critical role during learning and problem solving^4^,^5^. The relationship between cognitive estimation and EF has been established since the creation of the cognitive estimation task (CET). Shallice and Evans^1^ designed the CET as a tool to assess estimation abilities in patients with frontal lobe damage, an area of the brain associated with EF. They found that these patients provided significantly more extreme answers on the CET in comparison to the control participants, and therefore, concluded that the CET is an appropriate means of assessing EF.

Thus the primary function of the CET has been a clinical tool to assess EF for patients with neurological disorders^3^. Researchers have found deficits in CET performance in Alzheimer’s disease^6^,^7^, Korsakoff’s syndrome^8^, major depressive disorder^9^, and schizophrenia^10^. Few studies have examined CET performance in healthy populations, and even fewer have examined performance among children^2^,^11^,^12^,^13^.

## CET research with children

Previous research has found that children with pervasive developmental disorder^14^ and epilepsy^15^ had extreme CET performance in comparison to typically developing (TD) children. However, few studies have examined CET performance exclusively in TD children. In one example, CET performance was examined in a large sample of TD children 5–16 years old^13^. They found rapid development in CET performance between the ages of 5–9, after which the rate of development slowed down. The results were explained in light of EF development, which is consistent with CET research that classifies the CET as an EF task. However, more current research suggests that EF continues to develop into later adolescence^16^,^17^ and not only in childhood.

Other than EF, the developmental trajectory found in the previous study can be explained by educational factors. Young children have not learned units of measurement that are required in order to answer most of the CET questions. Quantity based questions have intuitively understood units, for the watermelon example above the unit is “seeds”, and young children can answer such questions, while other questions based on other content such as weight or time, require formal education of units of measurement in order to contemplate rational, possible answers. For example, “how much does a telephone weigh?” knowledge of units of weight is a prerequisite prior to problem solving. This distinction between the different types of CET questions was not considered in previous research and young childrens answers were scored as extreme, resulting in a scoring error; extreme scores in this case do not reflect poor CET performance.

In order to further our understanding of CET development we propose two modifications: 1) Examination of CET performance in TD children should be conducted after formal education of relevant units of measurement in order to produce meaningful results. 2) The CET questions should be categorized by content based on magnitude in order to examine how knowledge of units of measurement can effect performance for the different categories. Due to it’s predominant use as a clinical tool to assess EF abilities in neuropsychology patients, the CET has been assumed to be a cohesive construct, while other contributing factors such as question content and education have not been considered in CET research. Examination of CET performance in children provides a unique oppurtunity that can shed light on the relationship between factors effecting CET performance.

## The current study

The goal of the current study was to examine the typical development of CET performance. We compared 4^th^ graders, 6^th^ graders and adults CET performance. These grades were chosen based on Israel’s math curriculum to ensure that all participating children had learned relevant units of measurement, time and weight, at school. We predicted that children’s overall CET performance would be more extreme than adults, and that 4^th^ graders would have even more extreme scores compared to 6^th^ graders.

Our second goal was to make a contrast within the CET itself. The CET categories have been assumed to reflect one construct: the scores of the weight, time, and quantity questions have been grouped together in one CET score under the assumption that they measure the same ability^2^,^3^. Therefore, we examined the developmental trajectories for each CET category separately. Although the children that participated in the current study had learned the relevant units of measurement we predicted that the difference between them and adults’ in these categories would still be greater than the quantity category: children have less experience applying units of measurement in daily life and these questions may require greater EF involvement.

## Method

### Participants

The sample included 67 participants, including children and university students (see Table 1). All participants resided in the greater Jerusalem area, were native Hebrew speakers and did not have a diagnosis in learning disabilities (LD), attention deficit hyperactivity disorder (ADHD) or psychiatric disorders. Children were screened for diagnosis over the phone when the parents were called to schedule the experiment. In addition, the participants’ parents, and the university students completed demographic questionnaires that included questions regarding diagnostic history of psychiatric disorders, LD’s and ADHD. Two university students were not included in the analysis due to their diagnostic history. Five 4^th^ graders were removed from the final sample due to lack of understanding of weight and improper use of its units of measurement. The final sample included 30 university students, 15 4^th^ graders and 15 6^th^ graders.

The children were recruited through local e-mail lists for towns in the Jerusalem area. The children received prizes and the university students received monetary compensation or class credit for their participation. The experiment was approved by the ethics committee of the Seymour Fox School of Education at the Hebrew University of Jerusalem.

## Experimental procedure

### Children

The children’s experiment was conducted in their homes in a quiet room with a table, parents were not present in the room while the children completed the tasks. Informed consent was obtained from the participants’ parents’ who signed a consent form and completed a demographic questionnaire. The children completed three tasks in the following order: the CET, EF task (the Tower of Hanoi), and a non-symbol comparison task. The entire experiment lasted approximately 45 minutes. The same laptop computer was used for the computerized tasks, Tower of Hanoi and the non-symbolic comparison task.

### Adults

The university students signed informed consent, filled a demographic questionnaire and completed the same three tasks, in the same order as the children, in a laboratory at the Hebrew University in Jerusalem. The same laptop computer was used for the adults that was used for the children’s experiment. The experimental procedure for both children and adults was carried out in accordance with requirements of the ethics committee of the Seymour Fox School of Education at the Hebrew University of Jerusalem.

### The Cognitive Estimation Task

For the CET, the participants were instructed to provide their best possible estimate by providing a numerical answer, as opposed to a range or qualitative answer, with a unit of measurement for each question. For the children’s CET, the experimenter discussed the concepts of estimation and units of measurement to ensure the children understood and could properly apply the units (See Appendix 1 for the full CET introduction and instructions for children). An emphasis was placed on the units of weight because it is taught in Israel in the 4^th^ grade.

The university students were given an abridged version of the instructions. All participants (children and adults) were provided with a ‘units of measurements help sheet’ that included a list of units of measurement by category (Time: seconds, minutes, hours, days, weeks, months, years. Weight: grams, kilograms) that was accessible throughout the task. Quantity was not included on the help sheet because the unit was contingent on the question content e.g. seeds, cookies, tablespoons. The questions were asked in the same pseudorandomized order for all participants. For the CET, the experimenter placed a white card with the question in front of the participant and read the question aloud. Uniform help texts were provided for clarification if necessary (example: “How much does a wool coat weigh ? ” help text: a thick winter coat). The experimenter recorded the participant’s complete answer then moved on to the next question.

## Materials

### Creating the CET

In order to obtain valid and reliable results it is important that the participants CET performance be compared to their own culture^18^. To date, there are no norms for the CET in Hebrew, therefore, the questionnaire used in the current study was created for this experiment. The questionnaire was loosely based on the Biber CET (For further details see Bullard *et al*.^2^). Some of the original questions were directly translated into Hebrew, while others were modified to reflect Israeli culture and additional questions were added in order to examine each category sufficiently (see Supplementary Materials Table 1). The questionnaire included 36 questions divided equally into three categories: weight, time and quantity.

### CET pilot

We conducted a CET pilot with a sample of adults (N = 54, Age: M = 30.81 SD = 8.34) that were recruited by e-mail and social media. The participants completed the CET and demographic questionnaire over the internet, participants that reported a clinical history (LD’s, ADHD, neurological or psychological disorders) were removed from the analysis. To assess the reliability of our new CET questionnaire, we compared the raw scores of each CET question from the online sample to the university students and found no differences. The scores from the pilot were used in place of norms to calculate the scores of the current study. Supplementary Material Table 1 summarizes the questionnaire and descriptive statistics for the pilot sample.

### Scoring the CET

Prior to calculating scores, all the answers needed to be converted in order to unify units of measurement. Participants were instructed to answer each CET question with a numerical answer and a unit of measurement, therefore, the units varied within each question across participants (see Table 1 in Supplementary Materials for units). The scoring method for the CET must consider that there are many acceptable answers for each question. The Biber CET^2^ uses a binary scoring method based on percentile: participants’ answers that fall between the 5^th^ and 95^th^ percentile are scored as normal (for the watermelon example the normal range is 30–1000 seeds), answers above or below this range are scored as abnormal or bizarre.

Information is lost in the transformation of numerical answers into a categorical binary scoring system. We created a scoring method that takes into consideration the entire distribution of answers: absolute standard scores. The absolute standard score is a measure of extremeness that avoids the cancelling out of signed standard scores; similar to normed CET forms that measure extremeness without consideration of over or under-estimations. The answer to each CET question was converted into absolute standard scores using the data from the online pilot. The calculation is based on the formula for standard scores. For each CET question, the average answer from the pilot was subtracted from the participants answer, 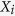, and divided by the pilot standard deviation (SD), the solution was then converted into absolute value.


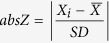


Four scores were created for each participant based on the absolute standard scores: the total CET score, the average of the absolute standard scores, and three category scores: weight, time and quantity, which was the average of the absolute standard scores within each category. Low CET scores were answers closer to the average reflecting superior CET performance, while higher scores reflect more deviation from the average and poorer CET performance. The advantage of using absolute standard scores over other scoring methods is that it allows for finer examination of differences between participants scores within the normative population.

### Control tasks

The CET has classically been used as a measure of EF, therefore, we included an EF task appropriate for children and adults as a control measure. In addition, CET performance involves numerical competence^19^, we choose to include a non-symbolic comparison task because it can easily administered across age groups, is not contingent on school curriculum and is related to math performance^20^.

### Tower of Hanoi

**Figure 1:**
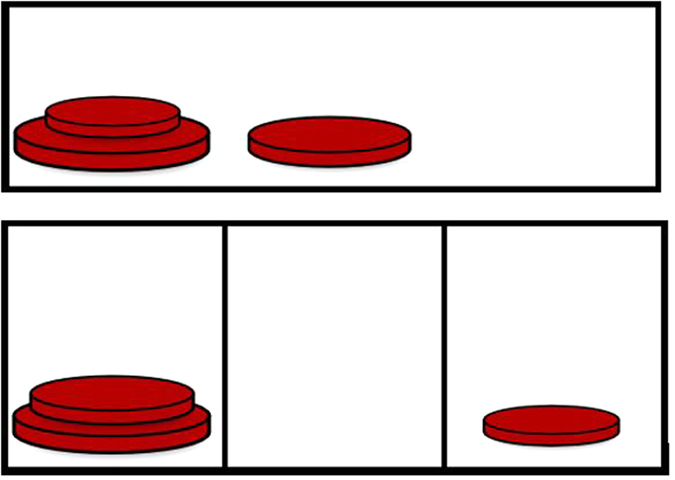
Tower of Hanoi.

For the EF task, the participants performed a computerized version of the Tower of Hanoi (TOH)^21^. The task included two configurations of disks on the top and bottom of the screen and each configuration consisted of three disks that varied in size: large, medium and small (see Fig. 1). The participants were instructed to move the disks that appeared on the bottom of the screen so they appeared the same as the disks on the top of the screen (target configuration) in as few moves as possible (Task from the Psychology Experiment Building Language- PEBL^22^). The TOH score was the average difference between minimum number of steps from the steps actually taken per trial. Lower scores indicate higher EF ability because the participant was able to finish the trial with fewer moves.

### Number sense task

The number sense task^20^ is a non-symbolic comparison task, using the PANAMATH program. On each trial, an array of spatially intermixed blue and yellow dots was presented on the computer screen. The participants’ task was to answer which color was more numerous by key press; however, the array was presented too rapidly to prevent serial counting (500 ms). The ratio between the two sets had several levels of difficulty that varied randomly across trials (ratios: 1:2, 3:4, 5:6, and 7:8). The number of dots in each trial ranged from 5–16 in each color and the color with more dots varied randomly. Half of the trials were controlled for area and half were controlled for dot size. Subjects performed one block that included 2 practice trials and 160 test trials. (For full details see Halberda, Mazzocco, & Feigenson^20^). A recent study exploring the psychometric properties of the non-symbolic comparison task found that Weber fraction is not the best measure of accuracy for non-symbolic comparison tasks, and therefore, we measured percent correct^23^.

## Results

For a summary of demographic information and performance on the control tasks see Table 1. Adults outperformed both groups of children in the TOH task (4^th^ grade: *t*(43) = 4.12, *p* < 0.001, *Cohen’s d* = 1.26, 6^th^ grade: *t*(43) = 3.98, *p* < 0.001, *Cohen’s d* = 1.21), but only 4^th^ graders for the number sense task (4^th^ grade: *t*(43) = −2.59, *p* < 0.05, *Cohen’s d* = −*0.79*; 6^th^ grade: *p* = 0.50). The two groups of children had comparable performance in TOH (*p* = 0.78), but for the number sense task the difference between groups of children approached significance (*p* = 0.05).

A mixed-design ANOVA was conducted to compare group performance on the CET. The between subject factor was age group (4^th^ graders/younger children, 6^th^ graders/older children and university students/adults) and the within subject factors were CET category (weight, quantity and time) and CET question (12 questions per category). Our new scoring method of the CET, unlike previous methods^2^ that used binary scores for each question, allowed us to add CET questions as a factor in the analysis, which validated our new questionnaire. We conducted the same ANOVA without CET question as a factor and received the same results we describe below (See Supplementary Materials Table 2 for a summary of this analysis).

A main effect for age group was found, *F*(2, 57) = 15.75 *p* < 0.001, *η*^*2*^ = 0.36. Planned comparisons revealed that adults total CET score (CET Total = 0.94), which measured CET performance across categories, was less extreme than both groups of children (4^th^ grade: CET Total = 1.90, *t*(44) = 4.57, *p* < 0.001, *Cohen’s d* = 1.38, 6^th^ grade: CET Total = 1.79, *t*(44) = 4.47, *p* < 0.001, *Cohen’s d* = 1.35). While no difference was found between the two group of children’s total CET score (*p* = 0.72). A significant interaction between CET category and age group was found, *F*(4, 110) = 3.70, *p* < 0.01, η2 = 0.11. To understand the source of the interaction we conducted three planned comparisons comparing the younger and older children’s performance in the CET category scores. No significant differences were found in any of the CET category scores between the groups of children (weight: *p* = 0.15; quantity: *p* = 0.79; time: *p* = 0.63).

**Figure 2:**
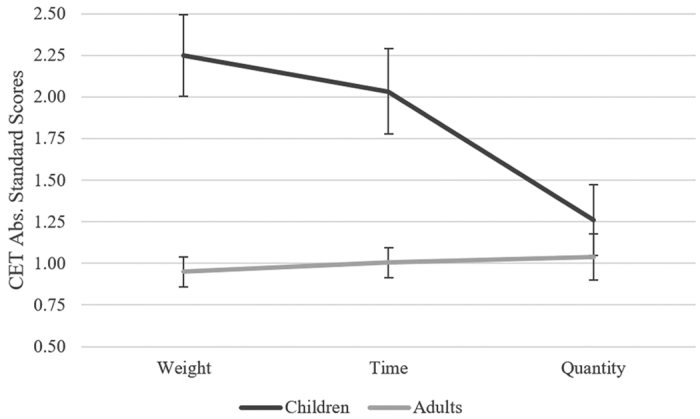
Comparison of adults and children’s CET performance by category. Children’s scores were overall more extreme than adults. There was no difference between age groups in the quantity category.

Children had comparable CET performance in both their total and category CET scores, and therefore, we conducted the same ANOVA as described above but with two age groups: children and adults. The interaction between CET category and age group remained significant *F*(2, 57) = 4.93, *p* < 0.01, η2 = 0.08. Planned comparisons revealed that children had significantly more extreme scores than adults in the weight category, *t*(58) = 5.27, *p* < 0.001, *Cohen’s d* = 1.38, and time category, *t*(58) = 3.96, *p* < 0.001, *Cohen’s d* = 1.04; no difference between groups was found in the quantity category (*p* = 0.21) (see Fig. 2). These comparisons suggest that despite the significant difference between children and adults overall CET performance, they had comparable performance in the quantity category. In addition, a significant interaction between CET question and age group was found, *F*(22, 37) = 3.80, *p* < 0.001, *η*^*2*^ = 0.06, and this interaction was modulated by category, *F*(22, 37) = 2.52, *p* < 0.001, *η*^*2*^ = 0.04. In order to explore the triple interaction, we conducted a planned comparison comparing the children and adults on the question level to ascertain if they had significantly different scores on particular CET questions, and if this differed by category. Nine questions yielded significant differences in the absolute standard scores between the children and adults, however, none remained significant using Bonferroni adjusted alpha levels.

The addition of the control tasks, TOH and number sense, to the model as covariance of non-interest, did not change the results of the current study all the main affects and interactions that were significant stayed significant after adding the covariance. Please see Table’s 3 and 4 in Supplementary Materials for a summary of these analyses.

To validate our scoring method of absolute standard scores we recalculated the CET scores based on the extreme binary scoring method, similar to the Biber CET, and repeated our main ANOVA using these scores. See Supplementary Materials for a description of how these scores were calculated. This analysis yielded similar results, there was a significant main effect for group *F*(2, 55) = 20.49, *p* < 0.001, *η*^*2*^ = 0.43 and interaction between CET category and age group *F*(4, 110) = 4.82, *p* < 0.001, *η*^*2*^ = 0.15.

## Discussion

Cognitive estimation is a unique clinical tool used to assess EF abilities for neuropsychology patients^3^ using everyday life situations; its questions lack exact, definitive answers, and the solution process requires multiple stages that involve numerous aspects of EF, such as working memory, attention and planning^1^. Due to its primary function as an assessment tool, research has not taken into account other skills required for CET performance or the possibility that the magnitude based categories require different skills or knowledge, such as units of measurement^2^,^11^,^13^. The absence of units of measurement is particularly significant when examining CET performance in children due to educational factors. Therefore, the main goal of the study was to examine CET development, while comparing performance between the CET categories.

Our study provides several contributions to CET research. This is the first study that statistically compared TD children and adult’s CET performance. We found children’s overall CET performance was more extreme than the adults, however, we found no differences in performance between the groups of children. We innovatively conducted a contrast within the CET. The calculation of one overall CET score masked differences in performance between magnitude based categories in children. Contrasts within the CET revealed that children and adults had comparable performance in the quantity category, while children’s performance in categories that required application of units of measurement, weight and time, was more extreme than adults. This suggests that the CET is not a unified construct and there are differences in the skills required to solve the different CET questions. In addition, we created a new scoring method that opens opportunities for future CET research in different cultures.

Research on CET development in healthy populations is limited^3^. Previous research has found rapid development in CET performance between the ages of 5–9, after which the rate of development slows down^13^,^14^. We found that 10 and 12-year-old children’s overall CET performance was significantly more extreme than adults. Taken together with previous research this suggests that although rate of development slows down in later childhood, CET performance is still not comparable to adults at the ages examined in the current study. We propose that the rapid development found in previous research in early childhood was due to lack of formal education of units of measurement, while our findings related to later childhood can be attributed to continuing EF development: prefrontal and parietal structures that support EF’s continue to develop into adolescence and early adulthood^16^.

Miyake *et al*.^4^ differentiated between three aspects of EF: 1) *inhibition*, the ability to override proponent or dominant responses, 2) *shifting*, the ability to move flexibly between mental sets or tasks, and 3) *updating*, the ability to monitor or maintain content in working memory. All three of these EF’s may be employed in different stages of CET performance. Participants need to *inhibit* irrelevant information and strategies, *shift* between question type and solution strategies, and *update* relevant information while storing and retrieving partial results.

A recent study examined the role of EF, based on the above perspective, on numerical estimation skills, and found that updating was the most important predictor of numerical estimation over shifting or inhibition^24^. Moreover, children with higher updating ability gained more from an EF training than those with low updating ability. The authors concluded that numerical estimation relies on the processing of multiple sources of information and, therefore, may be more dependent on updating skills. For example, the solution of the number line estimation task also has multiple unknown stages^25^ hence, updating may be the main EF for optimal CET performance.

The possible role of updating on CET performance can also explain our findings regarding CET categories. We found that children and adults had comparable performance in the quantity category, while children had significantly more extreme scores in the weight and time categories. The children in the current study had knowledge on the required units of measurement, and yet their scores were still more extreme than adults in these categories. This suggests that units of measurement serve as a prerequisite, without this knowledge a child could not even contemplate an answer, explaining the results of previous research^13^,^14^. However, the time and weight questions place a greater strain on EF, possibly updating, because there is more information to manage.

In contrast, for the solution of quantity questions, the participant primarily needs to select an appropriate numerical answer, the unit is given as part of the question, “how many seeds are in a watermelon ? ”, the unit “seeds” is provided and intuitive. Time and weight questions have an added level of difficulty, and possible greater involvement of the EF updating, in addition to providing a numerical estimate the participant needs to select an appropriate unit of measurement. The unit is not given as part of the question like in the quantity questions, adding an extra step and level of difficulty in the solution process. This places a greater strain on updating, the participant needs to monitor and maintain two sets of information: the numerical answer and the unit of measurement. In addition, within both sets of information multiple answers are appropriate, a range of numerical answers is correct and for several questions multiple units of measurement are correct as long the number is in agreement with the unit. This requires high levels of mental flexibility, which is difficult for children who are used to answering more general knowledge types of questions in school that have one, definitive answer.

In addition to EF, children have less experience applying units of measurement in everyday life situations, and their daily activities are different than adults. For the example, “how long does it take to paint a room ? ”, adults have life experience to back up their answers and provide an estimate. The fact that children can formulate responses in hours or days is irrelevant if they have no semantic framework to guide their answers. In addition, children may answer egocentrically or in reference to how long it would take a child to do something, for example, “How long does it take to swim across a pool at medium speed ? ”, children’s answers may be more extreme because they may use children’s speed as a reference.

One of the strengths of the CET is its applicability to everyday life situations, which is why it is used as an assessment of neurological rehabilitation. Solving CET questions involves a hybrid of skills including: semantic access, EF and estimation skills. In order to accurately assess all three aspects of CET performance in children, we propose new guidelines based on the results of the current study. 1) Children who have not learned units of measurement should not be tested on questions that require them. An extended CET form including only quantity questions can be created to more thoroughly examine CET performance in young children. Inclusion of magnitudes not included in the current study such as length and speed may be included only if the participating children have learned the relevant units of measurement. 2) Due to the importance of units of measurement, older children should have two separate scores: quantity and an average of the categories contingent on units of measurement. 3) We propose that a new CET form based on children’s everyday experiences be created in order to more accurately assess their cognitive estimation skills.

### Limitations

The current study examined CET development in a narrow age range and examined only one EF task and one numerical task. To further understand the relationship between CET performance and EF development future research should examine a broader age range from later childhood through early adulthood. We found no relationship between CET performance and the EF task in the current study. A larger variety of EF, numerical estimation, and math tasks should be included in future research to understand their involvement in CET performance. Future research can include other magnitude based categories such as speed, length and area, bearing in mind when children have learned these units in school. In addition, the current study did not include a formal education assessment, however, the children came from the same geographical area and similar SES.

## Conclusion

Cognitive estimation performance requires a hybrid of skills including basic mathematical knowledge, accessing relevant semantic frameworks and EF. The examination of CET development provided a deeper understanding of how these skills relate to one another. We found that children’s overall CET scores were more extreme than adults. Examination of the category scores revealed that adults and children in fact had comparable performance in the quantity questions, while children’s scores were more extreme than adults in the weight and time categories. These results have several implications for future CET research with children. 1) Knowledge of units of measurement is a prerequisite for CET performance, thereby, children should not be asked CET questions related to magnitudes for which they have not learned units of measurement. 2) EF development plays an important role in children’s CET performance and may play a role in the differential category performance. 3) Everyday life experiences is an important factor for CET performance, future research should consider creating CET questions with content based on children’s daily activities. The CET has the potential to serve as a tool to not only assess EF in children but also to examine how children acquire an understanding of different magnitudes and their respective units of measurement.

## Additional Information

**How to cite this article**: Silverman, S. and Ashkenazi, S. Deconstructing the Cognitive Estimation Task: A Developmental Examination and Intra-Task Contrast. *Sci. Rep.* **6**, 39316; doi: 10.1038/srep39316 (2016).

**Publisher's note:** Springer Nature remains neutral with regard to jurisdictional claims in published maps and institutional affiliations.

## Supplementary Material

Supplementary Material

## Figures and Tables

**Table 1:** Demographics and Performance on the Control Tasks.

N = 67	4^th^ grade	6^th^ grade	Adults
Age in years M (SD)	10.12 (0.32)	12.02 (0.56)	23.93 (1.62)
Gender N			
Female	13	7	20
Male	8	8	11
Tower of Hanoi M (SD)	2.25** (1.45)	2.41** (1.8)	0.91 (0.73)
Number Sense (% correct)	71.25* (6.48)	75.25 (4.08)	76.49 (6.37)

^*^p significant at the 0.01 level (2-tailed).

^**^p significant at the 0.001 level (2-tailed). Children were compared to adults.

Adults significantly outperformed both groups of children on the TOH task. Adults significantly outperformed 4^th^ graders on the number sense task.
