# Supplementary material for: Deconstructing the Cognitive Estimation Task: A Developmental Examination and Intra-Task Contrast
Source: Sci Rep. 2016 Dec 19;6:39316. doi: 10.1038/srep39316 (PMC5171918; doi:10.1038/srep39316)
Supplement: Supplementary Material [file srep39316-s1.pdf]

Deconstructing the Cognitive Estimation Task: A Developmental Examination and Intra-Task Contrast

Sarit Silverman* and Sarit Ashkenazi

Supplementary Material

SM Table 1 Descriptive Statistics of CET Raw Scores from the online pilot

Question*	Units	M	SD	5th percentile	95th percentile
How much does a cellphone weigh?	Grams	271.80	198.85	20.50	690.00
How much does a wool coat weigh?	Kilograms	2.29	1.49	0.41	5.00
How much does a two-seater couch weigh?	Kilograms	42.88	35.01	4.08	129.00
How much does a desktop computer weigh?	Kilograms	5.96	4.41	1.46	17.70
How much does a shopping cart weigh?	Kilograms	14.06	13.54	2.42	44.50
How much does a small folding table weigh?	Kilograms	6.21	5.10	1.00	19.90
How much does a plastic salad bowl weigh?	Grams	284.12	258.94	20.00	1000.00
How much does medium sized framed picture weigh?	Kilograms	2.78	2.30	0.35	7.95
How much does a falafel in a pita weigh?	Grams	447.10	311.41	40.50	1000.00
How much does a small washing machine weigh?	Kilograms	50.34	54.01	6.00	192.50
How much does one egg weigh?	Grams	52.88	39.65	5.75	100.00
How much does a dozen apples weigh?	Kilograms	2.71	1.56	1.01	5.92
How many slices of bread can be spread with chocolate spread until the container of spread is empty?	Slices	61.12	129.44	20.00	100.00
How many times can teeth be brushed from one tube of toothpaste?	Brushings	117.04	72.03	40.20	295.00
How many toffees are in a large package?	Candies	83.43	49.76	30.00	200.00
How many tablespoons of cottage cheese are in a standard package?	Tablespoons	13.12	7.35	4.45	29.25
How many flowers are on an almond tree when it blooms?	Flowers	6493.47	36345.22	53.90	7160.00
How many slices of bread are in a standard loaf?	Slices	22.76	5.25	15.00	30.00
How many tablespoons of sugar are there in a standard package?	Tablespoons	79.20	49.56	25.50	200.00
How many cookies fit in a cookie jar?	Cookies	39.15	23.64	15.10	100.00
How many hair-washings can be done from a large bottle of shampoo?	Hairwashings	51.10	31.18	13.40	118.00
How many people can be seated in a large soccer stadium?	People	29919.88	28169.18	1500.00	100000.00
How many sunflower seeds are in a small bag?	Seeds	609.80	1548.58	53.60	1950.00
How many sticks of spaghetti are in a half-kilo package?	Sticks	119.74	87.14	40.00	250.00
How long does it take fresh milk to spoil in the refrigerator?	Days	11.34	4.90	4.15	21.00
How long does it take to paint a medium sized room?	Hours	3.81	3.40	0.58	11.70
How long does it take one man to build a tent?	Minutes	18.73	14.38	5.00	60.00
How long does it take to boil water in an electric kettle?	Minutes	2.53	1.32	0.68	5.00
How long does it take to fill a bath with water?	Minutes	11.08	8.13	2.10	27.20

How long does it take to inflate an electric blow-up mattress?	Minutes	8.58	7.29	0.52	29.50
How long can flowers be in a vase until they wilt?	Days	6.12	3.29	2.00	14.00
How long does it take a carpenter to build a large closet?	Days	3.23	3.85	0.08	7.00
How long does it take to fill up a car with gas?	Minutes	4.12	2.13	2.00	10.00
How long does it take to swim across a pool at medium speed?	Minutes	2.46	2.60	0.33	9.70
How long does it take to blow up a balloon?	Seconds	34.05	25.10	5.75	88.80
How long does it take to build a shelf?	Minutes	17.34	15.21	1.61	60.00

*Questions in the study were in Hebrew, they were translated into English for the purpose of this paper.

SM Table 2 ANOVA excluding CET question from the analysis

	df	*F*	*p*	η^2
CET Category	2, 56	7.25	0.00	0.11
CET Category X Group	4, 114	3.70	0.01	0.12
Group	2, 57	15.75	0.00	0.36

SM Table 3 ANOVA with covariates of TOH and Number Sense, three age groups (4th graders, 6th graders, adults)

A three age groups

	df	*F*	*p*	η^2
Group	2, 55	12.97	0.00	0.32
CET Category	2, 54	0.00	0.99	0.00
CET Category X Group	4, 110	2.89	0.03	0.09
CET Question	11, 45	0.31	0.98	0.01
CET Question X Group	22, 92	4.41	0.00	0.14
CET Category X CET question	22, 34	0.59	0.93	0.01
CET Category X CET Question X Group	44, 70	2.48	0.00	0.08

SM Table 4 ANOVA with covariates of TOH and Number Sense, two age groups (children, adults)

	df	*F*	*p*	η^2
Group	1, 56	26.03	0.00	0.32
CET Category	2, 55	0.08	0.92	0.00
CET Category X Group	2, 55	3.44	0.04	0.06
CET Question	11, 46	0.20	1.00	0.00
CET Question X Group	11, 46	2.90	0.00	0.05
CET Category X CET question	22, 35	0.45	0.99	0.01
CET Category X CET Question X Group	22, 35	2.10	0.00	0.04

Comparison of absolute standard scores and percentile based scores

In order to compare our scoring method with the Biber scoring method, we recalculated our scores based on percentile from the online pilot data. Answers that were above the 95th percentile and below the 5th percentile were given a score of 0, and answers between the 5th and 95th percentiles were assigned a score of 1. We then calculated the sum of the scores within each category, such that higher scores reflect superior CET performance and low scores reflect poor performance. Please note that the Biber CET is based on norms from a sample of 113 participants and ours is based on a sample of 54 participants (which contributed to the rationale behind calculated absolute standard scores). Following the recalculated CET category scores, we conducted the same ANOVA as reported in the study, with the exception that this analysis did not examine CET performance on the question level due to the binary scoring method used in this analysis.
